# Brain O-GlcNAcylation in Neurodegenerative Diseases: Context-Dependent Mechanisms and Precision Therapeutic Translation

**DOI:** 10.3390/brainsci16080828

**Published:** 2026-08-04

**Authors:** Shaoshuai Lu, Ziyang Chen, Yanyan Wang, Hongsheng Bian, Shuang Yu, Lili Huang

**Affiliations:** College of Pharmacy, Heilongjiang University of Chinese Medicine, Harbin 150040, China; 13672286537@163.com (S.L.); 17367965055@163.com (Z.C.); wyy2891@126.com (Y.W.); bhs004@163.com (H.B.); 15504506203@163.com (S.Y.)

**Keywords:** O-GlcNAcylation, brain energy metabolism, synaptic plasticity, proteostasis, neurodegenerative diseases, OGA inhibitors, precision therapy

## Abstract

**Highlights:**

**What are the main findings?**

- O-GlcNAcylation exerts context-dependent effects in neurodegenerative diseases rather than being uniformly protective or detrimental.
- We propose a state-resolved framework integrating disease stage, cell type, subcellular localization, substrate specificity, and therapeutic intervention dynamics.

**What are the implications of the main findings?**

- This framework provides a more precise interpretation of conflicting findings regarding O-GlcNAc modulation across different neurodegenerative disorders.
- Future therapeutic strategies should focus on correcting disease-relevant O-GlcNAc states rather than on globally increasing or decreasing total O-GlcNAcylation.

**Abstract:**

O-linked β-N-acetylglucosamine modification (O-GlcNAcylation) is a dynamic, nutrient-sensitive post-translational modification that couples hexosamine biosynthesis pathway flux to protein function in neurons and glia. This reversible cycling, catalyzed by O-GlcNAc transferase (OGT) and O-GlcNAcase (OGA), integrates glucose, glutamine, acetyl-CoA, and nucleotide metabolism with synaptic activity, mitochondrial adaptation, transcriptional regulation, proteostasis, and neuroimmune signaling. Dysregulated O-GlcNAc cycling has been implicated in major neurodegenerative diseases, including Alzheimer’s disease (AD), Parkinson’s disease (PD), amyotrophic lateral sclerosis (ALS), frontotemporal dementia (FTD), and Huntington’s disease (HD), through effects on disease-related proteins, autophagy, mitochondrial function, and inflammatory networks. However, available evidence does not support a universal model in which global O-GlcNAc elevation is uniformly protective or global reduction is uniformly pathogenic. In this mechanistic narrative review, we integrate disease-specific and substrate-focused findings while distinguishing relatively mature translational evidence from model-based or hypothesis-generating observations. We propose a state-resolved framework in which disease-relevant O-GlcNAc states are interpreted across biological contexts, substrate/site specificity, and intervention dynamics. This framework helps reconcile divergent findings across experimental systems and highlights the limitations of indiscriminate global pathway modulation. Although OGA inhibitors represent the most advanced therapeutic strategy, their broad substrate effects underscore the need for pharmacodynamic biomarkers, human validation, brain-targeted delivery, and state-resolved approaches. Moving from bulk O-GlcNAc measurements toward precise correction of disease-relevant O-GlcNAc states across defined biological contexts will be essential for translating this biology into clinically meaningful interventions.

## 1. Introduction

Post-translational modifications (PTMs) regulate cellular signaling, gene expression, metabolic homeostasis, stress adaptation, and protein fate by altering protein conformation, activity, stability, localization, and interaction networks [1]. Among these modifications, O-linked β-N-acetylglucosamine modification (O-GlcNAcylation) is distinctive because it directly couples nutrient availability and cellular stress to protein function. O-GlcNAcylation involves the attachment of a single N-acetylglucosamine moiety to serine or threonine residues of predominantly nuclear, cytoplasmic, and mitochondrial proteins [2,3,4]. Unlike classical secretory-pathway glycosylation in the endoplasmic reticulum and Golgi apparatus, O-GlcNAcylation generally does not generate extended glycan chains and undergoes rapid and reversible cycling mediated by O-GlcNAc transferase (OGT) and O-GlcNAcase (OGA) [5,6,7].

The metabolic sensitivity of O-GlcNAcylation arises from its dependence on uridine diphosphate N-acetylglucosamine (UDP-GlcNAc), the end product of the hexosamine biosynthesis pathway (HBP). Because UDP-GlcNAc synthesis requires inputs from glucose, glutamine, acetyl-CoA, and uridine triphosphate (UTP) metabolism, the HBP integrates multiple dimensions of cellular nutrient status [8,9,10]. OGT uses UDP-GlcNAc to modify diverse protein substrates, whereas OGA removes the modification, allowing O-GlcNAc cycling to respond dynamically to nutrient availability, neuronal activity, and cellular stress [11,12,13]. This metabolic coupling is particularly relevant to the nervous system, where sustained synaptic transmission, long-distance axonal transport, local protein synthesis, mitochondrial trafficking, and long-term proteostasis impose exceptionally high and spatially heterogeneous energetic demands.

Accumulating evidence indicates that O-GlcNAcylation participates in multiple levels of brain function. In neurons, it regulates synaptic vesicle mobilization, receptor trafficking, postsynaptic density organization, mitochondrial positioning, activity-dependent transcription, and learning-related plasticity [14,15,16,17,18]. In glial cells, O-GlcNAc signaling may influence metabolic support, inflammatory activation, and maintenance of the neuronal microenvironment. These functions are further complicated by extensive crosstalk between O-GlcNAcylation and phosphorylation and by the compartmentalized architecture of neural cells. Consequently, identical changes in total O-GlcNAc abundance may reflect fundamentally different biological processes depending on the affected cell type, subcellular compartment, substrate protein, and modification site.

This biology is particularly relevant to neurodegenerative disease. Alzheimer’s disease (AD), Parkinson’s disease (PD), amyotrophic lateral sclerosis (ALS), frontotemporal dementia (FTD), Huntington’s disease (HD) share synaptic failure, mitochondrial dysfunction, impaired proteostasis, abnormal protein aggregation, oxidative stress, and chronic neuroinflammation [19,20]. O-GlcNAcylation intersects with each of these processes and modifies disease-relevant proteins, including tau, α-synuclein, TAR DNA-binding protein 43 (TDP-43), neurofilaments, and mutant huntingtin. The strongest disease-directed evidence currently comes from AD/tauopathy and PD, although its maturity differs substantially between these conditions. The evidence base for AD and related tauopathies includes pharmacological studies and clinical-stage evaluation of OGA inhibition, whereas the evidence base in PD remains supported predominantly by substrate-level and preclinical studies involving α-synuclein, dopaminergic-neuron stress, mitochondrial quality control, and microglial inflammation. Across both disease contexts, the direction and magnitude of benefit depend on the affected substrate, cell population, disease stage, and intervention schedule.

This context dependence creates both a challenge and an opportunity for therapeutic translation. Global OGA inhibition increases O-GlcNAcylation across a broad substrate landscape and has produced disease-relevant benefits in several preclinical models, particularly in tauopathy models. Recent microglial studies raise the clinically important possibility that the same intervention could combine protein-directed and anti-inflammatory effects. At the same time, broad modulation can influence autophagic flux, metabolic signaling, synaptic proteins, and peripheral tissues. The key translational task is therefore not to postpone development until absolute molecular specificity is available, but to define an exposure window in which beneficial neuronal and glial effects outweigh countervailing network effects.

Accordingly, this mechanistic narrative review integrates the metabolic basis, physiological functions, disease-associated mechanisms, therapeutic strategies, and analytical approaches used to study brain O-GlcNAcylation. Rather than treating all findings as equivalent, we distinguish relatively mature evidence supported by convergent findings from human tissues, disease models, pharmacological interventions, and clinical target-engagement studies from observations that remain predominantly model-based, substrate-focused, or hypothesis-generating. We further develop a context-dependent and state-resolved framework that emphasizes disease-specific substrates, cell-type- and compartment-sensitive mechanisms, intervention magnitude and duration, and the distinction between bulk O-GlcNAc abundance and substrate- or site-specific modification. This framework is used to evaluate current therapeutic strategies and to explain why future translation will likely require precise correction of disease-relevant O-GlcNAc states rather than indiscriminate global modulation of O-GlcNAc cycling.

## 2. Biochemical Basis of O-GlcNAcylation and Its Coupling to Brain Metabolism

### 2.1. The HBP–UDP-GlcNAc Axis as an Integrator of Metabolic Inputs

UDP-GlcNAc, the donor substrate for O-GlcNAcylation, is generated through the hexosamine biosynthesis pathway (HBP). Its synthesis requires inputs from glucose, glutamine, acetyl-CoA, and UTP metabolism, thereby linking O-GlcNAc signaling to multiple dimensions of cellular metabolic state [8,9,10]. Although only a fraction of glucose flux enters the HBP under many physiological conditions, changes in substrate availability and pathway activity can alter the intracellular UDP-GlcNAc pool and, consequently, influence O-GlcNAc cycling. This relationship, however, is not strictly linear, because cellular O-GlcNAc patterns are additionally shaped by OGT and OGA abundance and activity, substrate accessibility, protein–protein interactions, and subcellular localization [11,12,13,21,22,23,24,25,26].

The breadth of the O-GlcNAc proteome presents a fundamental problem of substrate selectivity. OGT and OGA regulate modification states across a large and diverse substrate landscape, yet individual proteins and sites respond differently to metabolic or enzymatic perturbation. Such selectivity is influenced by enzyme isoforms, catalytic and regulatory domains, adaptor or interacting proteins, local substrate concentration, protein conformation, and compartmentalization [21,22,23,24,25,26]. Accordingly, changes in total cellular O-GlcNAcylation should not be assumed to represent uniform changes across all substrates or functional pathways.

This issue is particularly relevant in the brain, where neurons and glial cells differ substantially in metabolic organization and functional demands. Neurons must coordinate synaptic activity with long-distance axonal transport, local translation, and spatially distributed mitochondrial energy supply, whereas astrocytes and microglia perform distinct roles in metabolic support, neurotransmitter homeostasis, immune surveillance, and inflammatory responses. In neurons, glucose-sensitive O-GlcNAc modification of the mitochondrial transport adaptor Milton has been shown to regulate mitochondrial motility, providing a direct mechanism by which nutrient status can influence organelle positioning [27]. More recently, activity-driven O-GlcNAcylation has been implicated in mitochondrial plasticity during neuronal activation [28]. Together, these findings support a functional connection between metabolic state, O-GlcNAc signaling, and neuronal energy adaptation.

Nevertheless, these observations should not be interpreted as evidence that global increases or decreases in O-GlcNAcylation produce equivalent effects across brain cell types. Because neurons, astrocytes, microglia, and oligodendrocytes differ in metabolic programs, substrate repertoires, and stress responses, similar changes in bulk O-GlcNAc abundance may reflect distinct or even opposing cell-specific processes. This cell-type dependence provides an important basis for interpreting apparently inconsistent findings across physiological and disease models [11,12].

### 2.2. O-GlcNAcylation and Phosphorylation: From Local Site Interplay to PTM Networks

O-GlcNAcylation and phosphorylation both frequently target serine and threonine residues and therefore exhibit extensive functional crosstalk [29,30,31,32,33,34,35,36,37,38]. Early models emphasized reciprocal occupancy at the same residue, where O-GlcNAc and phosphate are mutually exclusive. Although such direct competition occurs at selected sites, subsequent studies have demonstrated a substantially more complex relationship. O-GlcNAcylation at one residue can alter phosphorylation at nearby or distant sites by changing protein conformation, accessibility to kinases or phosphatases, subcellular localization, or protein–protein interactions [29,30,31,32,33]. In addition, O-GlcNAc cycling can regulate kinases, phosphatases, and signaling complexes themselves, thereby reshaping broader phosphorylation networks [34,35,36,37,38].

This distinction is important because global reciprocal changes in O-GlcNAcylation and phosphorylation do not necessarily indicate direct competition at individual residues. Depending on the substrate and cellular context, the two modifications may be mutually exclusive, spatially adjacent, sequentially coordinated, or indirectly coupled through regulatory enzymes and signaling networks. Therefore, mechanistic claims regarding O-GlcNAc–phosphorylation interplay require site-resolved evidence rather than inference from changes in total modification levels alone.

Tau provides one of the most extensively studied examples in neurodegeneration. Aberrant tau phosphorylation is a central feature of AD and related tauopathies, and reduced brain glucose metabolism has been associated with decreased O-GlcNAcylation and increased tau phosphorylation [39,40]. Experimental elevation of O-GlcNAcylation can reduce selected phospho-tau species and suppress tau aggregation in several disease models [41,42,43,44]. However, these findings should not be generalized into a universal one-to-one competition model across all tau residues. The functional relationship depends on the specific sites examined, the experimental system, and the magnitude and duration of O-GlcNAc modulation.

Beyond tau, proteins involved in neuronal signaling and plasticity—including Synapsin I, cAMP response element-binding protein (CREB), calcium/calmodulin-dependent protein kinase II (CaMKII), and glutamate receptor-associated proteins—are regulated within signaling environments in which O-GlcNAcylation and phosphorylation intersect [45,46,47,48,49,50,51,52,53,54]. These observations support a broader view of O-GlcNAc/phosphorylation crosstalk as a PTM network through which metabolic state can influence synaptic transmission, transcriptional responses, and proteostasis. A major future challenge is therefore to distinguish direct site competition from indirect network-level coupling and to determine which forms of crosstalk are causal in neurodegenerative disease.

### 2.3. Subcellular Compartmentalization Shapes Functional Output

O-GlcNAc cycling is spatially organized rather than uniformly distributed across the cell. OGT and OGA act within distinct molecular environments, and the functional consequences of O-GlcNAcylation depend partly on where the relevant enzymes, substrates, and interacting partners are localized. In the nucleus, O-GlcNAcylation is extensively associated with transcriptional regulation, chromatin-related processes, circadian control, and stress-responsive signaling [50,51,52,53,55,56]. In the cytoplasm, O-GlcNAc modification affects signaling proteins, cytoskeletal components, synaptic machinery, and protein complexes involved in receptor trafficking and local translation [27,28,45,46,47,48,49,57,58,59,60,61,62,63,64]. Mitochondrial and mitochondria-associated O-GlcNAc signaling has also been linked to bioenergetic regulation, organelle transport, and oxidative-stress responses [27,28,65].

The polarized architecture of neurons makes this spatial organization especially important. Axons, presynaptic terminals, dendritic shafts, dendritic spines, and somatic compartments differ in protein composition, energetic demand, calcium dynamics, and local signaling environments. For example, O-GlcNAc-dependent regulation of Milton provides a mechanism linking metabolic state to mitochondrial trafficking along neuronal processes [27], whereas activity-driven changes in neuronal O-GlcNAcylation have been associated with mitochondrial adaptation [28]. At postsynaptic sites, O-GlcNAc-modified proteins are enriched within complex signaling assemblies, further indicating that local modification states may influence synaptic organization and plasticity [60,61,62,63].

However, current evidence does not yet establish that all neuronal compartments maintain independently regulated O-GlcNAc “microenvironments” in a quantitatively defined manner. Such a model is biologically plausible but remains incompletely resolved because most studies rely on bulk tissue, whole-cell measurements, or limited subcellular fractionation. Similarly, mitochondrial O-GlcNAc biology requires careful interpretation because mitochondrial localization, mitochondria-associated proteins, and indirect cytosolic regulation of mitochondrial function are not experimentally equivalent.

Accordingly, mechanistic interpretation should prioritize the affected cell type, subcellular compartment, substrate, and modification site rather than total O-GlcNAc abundance alone. The analytical approaches required to distinguish these levels are discussed in Section 2.4.

### 2.4. Methodological Approaches for Measuring O-GlcNAc States in Neural Tissues

Interpretation of O-GlcNAc biology in neural tissues requires a clear distinction among total O-GlcNAc abundance, OGT and OGA expression, enzymatic cycling activity, modification of a defined substrate, and occupancy of an individual serine or threonine site. These endpoints are not interchangeable. Pan-O-GlcNAc antibodies such as RL2 and CTD110.6 are widely used for immunoblotting, dot blotting, immunohistochemistry, and immunofluorescence, but they recognize partially different epitope repertoires, and the resulting signals can be influenced by fixation, extraction, transfer conditions, glycan competition, and the metabolic state of the sample. CTD110.6 can also cross-react with proteins bearing N-linked GlcNAc-containing structures under severe glucose deprivation [66]. Conclusions based on a single pan-O-GlcNAc antibody should therefore be supported, where feasible, by free-GlcNAc competition, enzymatic removal of O-GlcNAc, genetic or pharmacological manipulation of OGT or OGA, a second antibody with a different recognition profile, or an orthogonal chemoenzymatic or mass-spectrometric method.

Measurements of OGT and OGA protein abundance provide useful contextual information but do not directly report catalytic flux. Enzyme isoforms, subcellular localization, interacting proteins, substrate accessibility, UDP-GlcNAc availability, and compensatory feedback can uncouple enzyme abundance from total or substrate-specific O-GlcNAcylation. Claims of altered O-GlcNAc cycling are therefore strengthened by combining OGT and OGA immunoblotting with enzyme-activity assays, measurements of UDP-GlcNAc or related metabolic inputs, and time-resolved assessments of modification gain or loss following pathway perturbation.

Protein-specific analysis is commonly performed by immunoprecipitating a candidate substrate and probing for O-GlcNAc, or by enriching O-GlcNAc-modified proteins and probing for the substrate. Reciprocal immunoprecipitation increases confidence, but the results remain sensitive to antibody specificity, epitope masking, co-precipitating proteins, detergent conditions, fractionation, and the typically low stoichiometry of O-GlcNAcylation. The modified signal should be normalized to the amount of immunoprecipitated substrate and interpreted together with total substrate abundance and its distribution between soluble and insoluble fractions. Substrate- or site-specific O-GlcNAc antibodies are especially promising because they can convert a global pathway readout into a pharmacologically useful proteoform measurement. A rabbit monoclonal antibody recognizing tau O-GlcNAcylated at Ser400 has demonstrated that a defined O-GlcNAcylated tau proteoform can be detected in vitro and in vivo [67]. Such reagents could support mechanistic studies and pharmacodynamic monitoring, particularly when validated against targeted mass spectrometry. Their current limitation is scarcity: each antibody requires stringent validation against unmodified and phosphorylated peptides, site-mutant proteins, enzymatic removal of O-GlcNAc, and disease-relevant tissue controls.

Chemoenzymatic labeling, lectin-based enrichment, and mass spectrometry provide broader and more site-resolved coverage. Chemoenzymatic histology can reveal the tissue and cellular distribution of O-GlcNAc without relying solely on pan-O-GlcNAc antibody recognition [68]. Quantitative human-brain proteomics using tandem-mass-tag labeling, chemoenzymatic photocleavage enrichment, and liquid chromatography–mass spectrometry has identified more than one thousand O-GlcNAc sites and demonstrated that AD-associated changes can be resolved at the peptide level [69]. However, O-GlcNAc is labile and often substoichiometric; enrichment is usually required, site localization may depend on electron-based fragmentation methods, and modified-peptide abundance should be normalized to the corresponding parent protein whenever possible [61,62,63]. Failure to distinguish altered protein abundance from altered site occupancy can otherwise lead to incorrect mechanistic conclusions.

Postmortem brain studies introduce additional sources of variation. Brain region, neuropathological stage, postmortem interval, agonal state, fixation or freezing history, metabolic comorbidities, extraction buffer, soluble versus insoluble fractionation, and failure to adequately suppress OGA and phosphatase activities during sample processing can all influence the apparent relationships among total O-GlcNAc, O-GlcNAcylated tau, and phosphorylated tau. Neuronal loss and glial expansion further alter bulk-tissue composition. Consequently, apparently discordant reports of increased or decreased O-GlcNAc signals in AD tissue need not represent true biological opposition. A lower O-GlcNAc-to-tau ratio may reflect reduced site occupancy, altered total tau abundance, selective loss of a neuronal population, redistribution of tau into an insoluble fraction, or technical loss of the modification during processing. Conversely, a higher bulk signal may reflect changes in cellular composition, including glial expansion, compensatory reductions in OGA abundance or activity, or increased modification of proteins other than tau. Co-staining with cell-type markers, laser-capture microdissection, sorted nuclei or cells, spatial proteomics, and cell-type-specific immunoprecipitation can reduce—but not eliminate—these ambiguities.

At minimum, tissue-based studies should report brain region and disease stage, postmortem interval and sample preservation, extraction and fractionation procedures, inclusion of OGA and phosphatase inhibitors, antibody clone and validation controls, loading and parent-protein normalization, and whether the endpoint represents abundance, site occupancy, or cycling activity. A practical evidence hierarchy is: (i) a global or spatial screen; (ii) reciprocal immunoprecipitation or enrichment-based substrate analysis; (iii) site-resolved confirmation using a validated antibody or mass spectrometry; and (iv) genetic or pharmacological perturbation demonstrating functional reversibility. This tiered approach is particularly important when a change in O-GlcNAcylated tau or another disease-associated protein is used to support a causal or therapeutic claim.

**Figure 1:**
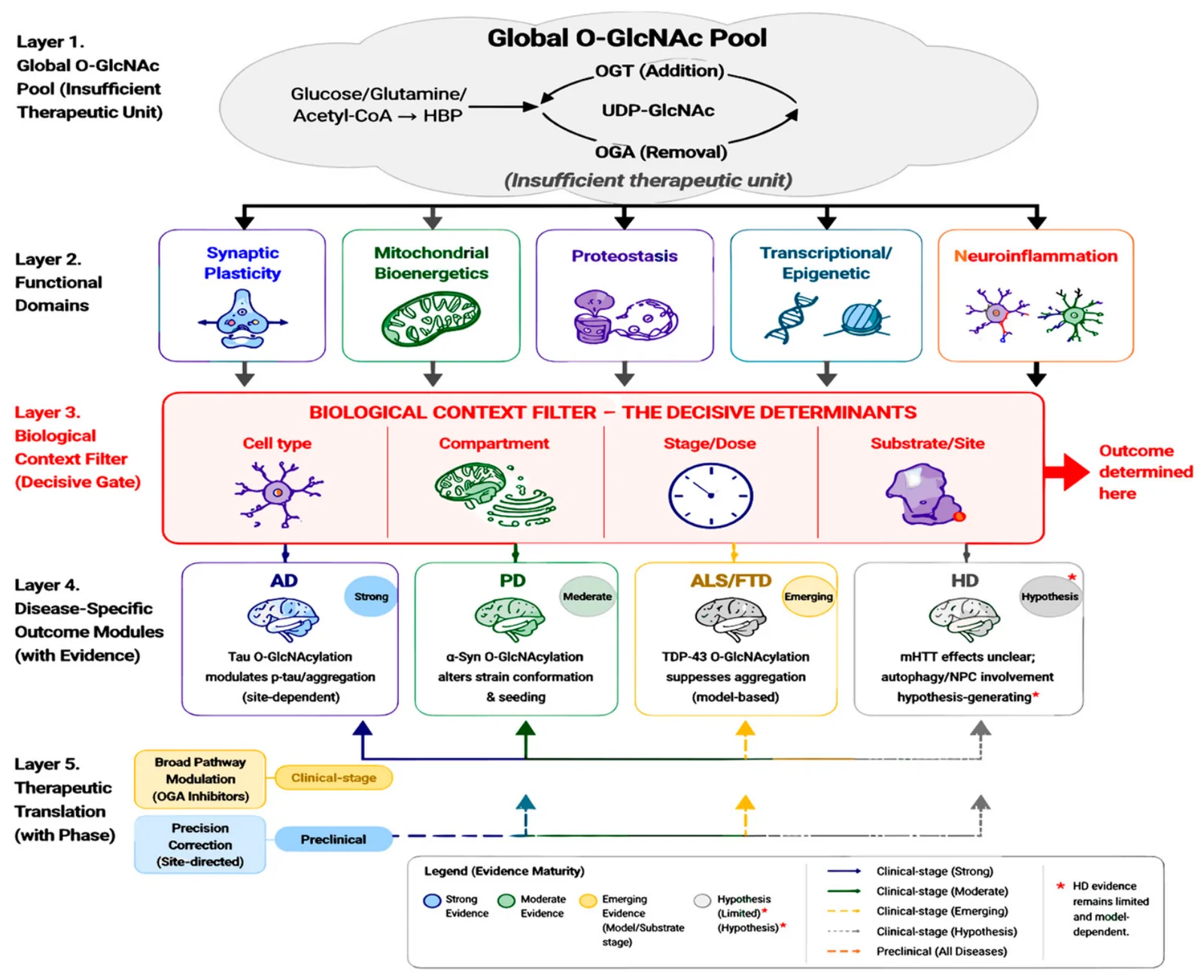
State-resolved framework linking brain O-GlcNAc metabolism to disease outcomes and therapeutic strategies. Metabolic inputs converge through the HBP to generate UDP-GlcNAc; OGT adds and OGA removes O-GlcNAc. Five layers are shown: (1) the global O-GlcNAc pool; (2) functional domains; (3) context determinants (cell type, compartment, stage/dose, and substrate/site); (4) disease-specific modules with graded evidence maturity (strong, moderate, emerging, or hypothesis-generating); and (5) broad pathway modulation versus site-directed precision correction. Outcomes vary with biological context and intervention dynamics, supporting correction of disease-relevant O-GlcNAc states rather than indiscriminate global modulation. Abbreviations: AD, Alzheimer’s disease; ALS, amyotrophic lateral sclerosis; FTD, frontotemporal dementia; HBP, hexosamine biosynthesis pathway; HD, Huntington’s disease; mHTT, mutant huntingtin; NPC, nuclear pore complex; O-GlcNAc, O-linked β-N-acetylglucosamine; OGA, O-GlcNAcase; OGT, O-GlcNAc transferase; PD, Parkinson’s disease; p-tau, phosphorylated tau; TDP-43, TAR DNA-binding protein 43; UDP-GlcNAc, uridine diphosphate N-acetylglucosamine; α-Syn, α-synuclein.

A comprehensive visual overview of this multilevel framework, spanning metabolic input, functional domains, contextual determinants, disease outcomes, and therapeutic strategies, is provided in Figure 1.

## 3. Physiological Functions of O-GlcNAcylation in the Brain

### 3.1. Neurogenesis and Neuronal Maturation

Neurogenesis and neuronal maturation require coordinated regulation of neural stem and progenitor cell proliferation, lineage commitment, differentiation, migration, neurite extension, and the establishment of axodendritic polarity. O-GlcNAcylation is dynamically regulated during neural development and has been implicated in several of these processes [70]. However, the available evidence spans distinct developmental stages and experimental systems, and the specific contribution of individual O-GlcNAc-modified substrates remains incompletely resolved.

Genetic studies provide strong evidence that O-GlcNAc cycling is essential for mammalian development. Complete loss of OGT causes embryonic lethality in mice, and Ogt-dependent protein O-GlcNAcylation is required for somatic cell function and embryonic viability [71,72]. These findings establish the fundamental biological importance of OGT but should not, by themselves, be interpreted as direct evidence for a neuron-specific developmental mechanism. More specific studies suggest that O-GlcNAc signaling intersects with developmental regulators and pathways involving Notch, signal transducer and activator of transcription 3 (STAT3), Sox2, and Gli2 [70,73]. Nevertheless, the direction and functional consequences of these interactions vary across cell types and developmental contexts, highlighting the need to distinguish general developmental dependence on OGT from substrate- and lineage-specific mechanisms.

Human genetic evidence further supports the importance of O-GlcNAc homeostasis in nervous-system development. Pathogenic variants in OGT have been associated with X-linked intellectual disability, and functional studies indicate that disease-associated variants can impair OGT catalytic activity or perturb cellular O-GlcNAc regulation [74,75,76]. These observations provide an important bridge between molecular O-GlcNAc biology and human neurodevelopmental phenotypes. However, the mechanisms linking individual OGT variants to specific neuronal, synaptic, or circuit-level abnormalities remain incompletely defined.

O-GlcNAc signaling may also participate in the differentiation and functional maturation of non-neuronal brain cells, including astrocytes and oligodendrocytes [11,12,70]. Such effects could indirectly influence neuronal development through metabolic support, extracellular homeostasis, and myelination. At present, however, the evidence base is less developed than that for global developmental dependence on OGT, and cell-type-resolved causal studies remain limited.

Collectively, current evidence establishes O-GlcNAc cycling as an essential component of neural development, although the underlying mechanisms vary across developmental stages and cell lineages. Lineage-specific genetic manipulation combined with site-resolved O-GlcNAc mapping will be necessary to identify the substrates that regulate neurogenesis and neuronal maturation and to distinguish developmental functions from disease-associated changes in the adult brain.

### 3.2. Synaptogenesis and Synaptic Plasticity

Synaptic plasticity depends on coordinated regulation across presynaptic terminals, postsynaptic signaling assemblies, local metabolic systems, and activity-dependent transcriptional programs [14,15,16]. O-GlcNAcylation contributes to several of these processes, but the strength of evidence differs substantially among substrates and mechanisms. Some proteins have been mapped to specific O-GlcNAc sites with functional validation, whereas others are linked to O-GlcNAc signaling primarily through proteomic association or broader pathway-level evidence. Distinguishing these evidence levels is essential for interpreting the role of O-GlcNAcylation in synaptic organization.

At presynaptic sites, Synapsin I provides one of the clearest examples of site-resolved functional regulation. O-GlcNAc sites on Synapsin I occur within domains involved in protein interactions and in proximity to phosphorylation-regulated regions [45]. In particular, O-GlcNAcylation at Thr87 has been shown to regulate Synapsin I localization and the size of the reserve pool of synaptic vesicles [46]. These findings provide direct evidence that a defined O-GlcNAc site can influence presynaptic vesicle organization. Beyond Synapsin I, heterogeneous nuclear ribonucleoprotein R (hnRNP R) interacts with OGT and promotes O-GlcNAcylation of eukaryotic translation initiation factor 4 gamma (eIF4G), thereby facilitating axonal protein synthesis [59]. O-GlcNAc modification of the mitochondrial transport adaptor Milton also links nutrient-sensitive signaling to mitochondrial motility and positioning along neuronal processes [27], while activity-driven O-GlcNAcylation has been implicated in mitochondrial adaptation during neuronal activation [28]. Together, these mechanisms suggest that presynaptic and axonal O-GlcNAc signaling can coordinate vesicle availability, local translation, and energy distribution.

Postsynaptically, proteomic studies have identified numerous O-GlcNAc-modified proteins in postsynaptic density preparations [60,61,62,63], indicating that O-GlcNAc signaling is embedded within synaptic protein networks. However, proteomic detection alone does not establish that modification of a given protein is functionally required for synaptic plasticity. Among the better-characterized examples, O-GlcNAcylation of the α-amino-3-hydroxy-5-methyl-4-isoxazolepropionic acid (AMPA) receptor subunit GluA2 has been associated with a distinct form of hippocampal long-term depression [48,64], and OGT has been shown to regulate excitatory synapse maturation [49]. More recent evidence implicating O-GlcNAc regulation of the N-methyl-D-aspartate receptor (NMDAR) subunit GluN1 further suggests that receptor-associated O-GlcNAc signaling may contribute to disease-related synaptic dysfunction [54].

An additional level of regulation involves the organization of postsynaptic signaling assemblies. The postsynaptic density (PSD) is a highly dynamic molecular network in which scaffold proteins and signaling components undergo multivalent interactions and, in some cases, liquid–liquid phase separation [77,78,79,80,81,82,83]. Direct evidence has shown that O-GlcNAcylation can modulate phase separation of the synaptic GTPase-activating protein (SynGAP)/postsynaptic density protein 95 (PSD-95) system [78], providing a mechanistic link between a specific PTM and the mesoscale organization of postsynaptic complexes. By contrast, SH3 and multiple ankyrin repeat domains protein (SHANK), guanylate kinase-associated protein (GKAP), Homer, and related scaffold proteins are well-established organizers of PSD architecture [79,80,81,82,83], but their inclusion in an O-GlcNAc-centered model should be interpreted cautiously unless direct modification and functional consequences have been demonstrated. These proteins therefore provide relevant structural context for PSD organization rather than equivalent evidence of direct O-GlcNAc-dependent regulation.

Current evidence links O-GlcNAcylation to presynaptic vesicle organization, axonal translation, mitochondrial positioning, receptor-associated plasticity, and postsynaptic complex assembly. However, direct site-specific evidence is available for only a limited number of substrates. Future studies should therefore prioritize endogenous site perturbation and in vivo validation to distinguish causal synaptic mechanisms from broader associations with neuronal state.

### 3.3. Learning, Memory, Circadian Rhythms, and Stress Responses

The contribution of O-GlcNAcylation to higher-order brain function extends beyond local synaptic regulation to activity-dependent transcription, memory formation, circadian timing, and stress-responsive signaling. These processes operate across different temporal scales and cell types, and their integration through O-GlcNAc cycling provides a potential mechanism by which metabolic state can influence neural adaptation.

Long-term memory formation requires coordinated signaling from activated synapses to the nucleus and the induction of transcriptional programs that stabilize persistent changes in neuronal function [50,51]. CREB is a central component of these programs. O-GlcNAcylation of CREB has been shown to regulate its transcriptional activity, and modification at Ser40 can influence CREB-dependent gene expression and neuronal phenotypes [52,53]. Together with evidence that dysregulated hippocampal O-GlcNAcylation impairs synaptic plasticity and memory [17], these findings support a role for O-GlcNAc cycling in coupling cellular state to memory-related transcription. Nevertheless, the consequences of altering global O-GlcNAc levels cannot be assumed to arise solely through CREB, because multiple synaptic and nuclear substrates are modified simultaneously.

O-GlcNAc signaling also intersects with circadian regulation. Core clock components, including brain and muscle ARNT-like protein 1 (BMAL1) and circadian locomotor output cycles kaput (CLOCK), are influenced by O-GlcNAc-dependent mechanisms, and the HBP and O-GlcNAc-processing enzymes contribute to daily rhythms in protein O-GlcNAcylation [55,56]. These observations support bidirectional coupling between metabolic state and circadian timing. In the nervous system, such coupling may be relevant to sleep–wake regulation, synaptic homeostasis, and cognitive performance, although direct causal links between specific clock-protein O-GlcNAc sites and neurodegenerative phenotypes remain incompletely established.

Stress and inflammatory responses represent another context in which O-GlcNAc effects are strongly cell-type- and disease-dependent. At the physiological level, O-GlcNAcylation has been linked to inflammatory signaling networks involving nuclear factor kappa B (NF-κB)-related pathways and inflammasome regulation, but the direction of these effects varies with cell state, stimulus, and experimental system. Emerging evidence in disease models suggests that cell-type-specific O-GlcNAc reprogramming—particularly in microglia—may modulate inflammatory states [84]. The pathological implications of these observations are discussed in detail in Section 4.1 and Section 4.2.

Collectively, these findings link O-GlcNAc cycling to memory-related transcription, circadian regulation, stress adaptation, and neuroimmune signaling. Future studies should determine which cell-specific O-GlcNAc events causally regulate these processes and which instead represent compensatory responses or downstream markers of altered brain states.

Representative substrates, functional implications, and key evidence boundaries across subcellular compartments and cell types are summarized in Table 1.

## 4. O-GlcNAc Imbalance in Neurodegenerative Diseases

Neurodegenerative diseases share several pathological features, including synaptic dysfunction, abnormal protein aggregation, mitochondrial impairment, disrupted autophagy–lysosome and proteasome systems, oxidative stress, and chronic neuroinflammation [19,20]. O-GlcNAcylation intersects with many of these processes, but its mechanistic and translational significance differs substantially across diseases. Importantly, evidence should not be considered equivalent merely because an O-GlcNAc-related change has been observed. Human tissue associations, genetic perturbation studies, pharmacological interventions in disease models, site-specific protein chemistry, and pathway-level observations provide different levels of causal and translational support.

The maturity of evidence varies markedly across diseases. AD and related tauopathies are supported by human tissue studies, mechanistic experiments, pharmacological interventions, and clinical-stage evaluation. PD evidence is derived primarily from substrate-specific and preclinical studies, whereas findings in ALS/FTD remain largely model- and substrate-based. Evidence in HD is comparatively limited and should currently be regarded as hypothesis-generating [39,40,41,42,43,44,65,84,85,86,87,88,89,90,91,92,93,94,95,96,97,98,99,100,101,102,103,104,105,106,107,108,109,110,111,112,113,114]. The following sections assess each disorder according to both its proposed mechanisms and the strength of supporting evidence.

### 4.1. Alzheimer’s Disease: From Metabolic Dysfunction to Tauopathy, Mitochondrial Impairment, and Neuroinflammation

Among neurodegenerative diseases, AD provides the most mature body of evidence linking dysregulated O-GlcNAc homeostasis to disease-relevant molecular and cellular phenotypes. A central observation is the relationship among impaired brain glucose metabolism, reduced O-GlcNAcylation, and tau pathology. Human and experimental studies have linked reduced glucose utilization to lower protein O-GlcNAcylation and increased tau phosphorylation [39,40]. Because tau contains multiple phosphorylation and O-GlcNAc modification sites, these findings have supported a model in which metabolic dysfunction may shift tau toward pathological modification states. However, this relationship should not be interpreted as universal reciprocal competition between O-GlcNAcylation and phosphorylation at all tau residues, because the effects are site-, model-, and context-dependent.

Experimental studies provide the strongest causal support for therapeutic modulation in AD. Selective OGA inhibition with research compounds such as Thiamet-G and more advanced brain-penetrant inhibitors increases brain O-GlcNAcylation and has been associated with reductions in selected phospho-tau species, tau aggregation, neuronal loss, cerebrospinal fluid tau concentrations, and brain volume loss in multiple complementary tauopathy models [43,44,91]. The rTg4510 study of MK-8719 is particularly informative because oral dosing produced concordant central and peripheral pharmacodynamic responses, evidence of target engagement, reductions in pathological tau, and attenuation of forebrain volume loss [91]. These studies do not establish that every tau site or every stage of AD will respond identically, but they demonstrate that OGA inhibition can produce sustained disease-relevant effects rather than merely altering a biochemical marker. The translational implications of these findings, including their potential relationship with microglial inflammation, are discussed further below.

O-GlcNAc-related mechanisms in AD extend beyond tau. O-GlcNAcylation has been implicated in amyloid precursor protein (APP) processing, including the promotion of non-amyloidogenic processing in experimental systems [42]. Mitochondrial dysfunction represents another mechanistic axis. Reduced O-GlcNAcylation of mitochondrial proteins, including ATP5A, has been associated with impaired ATP synthase activity in AD-related contexts [65], supporting a link between dysregulated O-GlcNAc signaling and bioenergetic failure. These observations are particularly relevant because synaptic maintenance depends on spatially coordinated mitochondrial function and energy supply. However, changes in mitochondrial protein O-GlcNAcylation should be distinguished from global cellular O-GlcNAc changes and from indirect effects of cytosolic signaling on mitochondrial function.

Neuroinflammation provides a second and potentially complementary therapeutic mechanism. Kim and colleagues combined analyses of postmortem human hippocampal tissue, an intracerebroventricular lipopolysaccharide (LPS)-induced neuroinflammation model exhibiting AD-relevant cognitive and neuronal abnormalities, and cultured microglial cells [84]. The human analysis included samples from three patients with AD and three controls and showed lower global and microglia-associated O-GlcNAc signals, together with increased levels of inflammatory markers associated with NF-κB and NOD-like receptor family pyrin domain-containing 3 (NLRP3). In the mouse model, glucosamine administration restored O-GlcNAcylation, increased O-GlcNAc modification of the NF-κB subunits p65 and c-Rel, limited their nuclear accumulation, reduced inflammatory signaling, shifted microglial marker profiles away from a pro-inflammatory state, and improved memory and neuronal survival. These findings are consistent with a mechanism through which O-GlcNAc enhancement may complement tau-directed effects by attenuating maladaptive microglial inflammation.

The translational interpretation nevertheless requires restraint. The human sample was small, and the LPS model primarily represents inflammation-driven neural injury rather than the complex amyloid and tau pathology of sporadic AD. Confirmation in amyloid- and tau-based models, patient-derived multicellular systems, and models involving microglia-specific manipulation of OGT or OGA will be required to determine whether the same mechanism operates across disease stages. Separately, chronic sleep deprivation has been linked to dysregulated whole-brain O-GlcNAc homeostasis and aggravated AD-like pathology [87]. This observation is complementary but does not establish the same microglial mechanism.

AD and related tauopathies currently provide the strongest rationale for O-GlcNAc-directed intervention. However, preclinical effects on tau proteoforms, neurodegeneration, and microglial inflammatory signaling have not yet been demonstrated concurrently in the same amyloid–tau model or in patients. The negative PROSPECT-ALZ findings further show that central OGA engagement and biomarker modulation do not necessarily translate into clinical benefit [92,114]. Future studies should therefore define exposure ranges that favor disease-relevant tau and neuronal or glial states while preserving physiological O-GlcNAc signaling.

### 4.2. Parkinson’s Disease

PD is characterized by progressive degeneration of nigrostriatal dopaminergic neurons, α-synuclein-rich pathological inclusions, mitochondrial dysfunction, and disruption of interconnected protein- and organelle-quality-control systems [93,95,115]. O-GlcNAc biology may contribute to PD at several separable but potentially convergent levels, including defined α-synuclein proteoforms, dopaminergic-neuron survival, mitochondrial quality control, autophagic and lysosomal flux, mitophagy, and microglial inflammatory signaling. Together, substrate-level studies, toxin-based disease models, observations in human tissue, and recent investigations of microglial responses provide a stronger therapeutic rationale than would be inferred from measurements of total O-GlcNAc abundance alone [85,86,93,94,95,96,97,98,99,115,116].

At the substrate level, α-synuclein provides a particularly informative example of site-specific O-GlcNAc regulation in neurodegeneration. Chemically and biochemically defined studies have shown that O-GlcNAc modification at specific residues can alter α-synuclein aggregation kinetics, toxicity, monomer dynamics, fibril conformation, seeding, and pathological propagation [94,96,99]. O-GlcNAcylation has also been shown to favor an amyloid strain with reduced seeding competence and pathological activity [96]. Importantly, these effects are not adequately captured by measurements of total cellular O-GlcNAcylation. Instead, they demonstrate that modification of a defined disease-associated protein at particular sites can reshape its biophysical properties and pathological behavior.

The relationship between O-GlcNAcylation and autophagy requires more cautious interpretation. O-GlcNAc signaling has been linked to autophagic regulation and α-synuclein homeostasis [97,98], but the available evidence does not support a universal model in which increasing O-GlcNAcylation necessarily impairs pathological protein clearance. Pharmacological OGA inhibition has been reported to enhance autophagy in the brain through an mechanistic target of rapamycin (mTOR)-independent mechanism [97], whereas other experimental contexts indicate more complex interactions among O-GlcNAc cycling, autophagic pathways, lysosomal function, and α-synuclein homeostasis [98]. These apparently divergent findings suggest that the outcome may depend on cell type, disease model, basal autophagic capacity, duration and magnitude of pathway modulation, and the specific molecular nodes affected.

Mitophagy provides a more specific, although currently indirect, link between O-GlcNAc signaling and PD-related mitochondrial quality control. The PTEN-induced kinase 1 (PINK1)–Parkin pathway is a central mechanism for recognizing and eliminating damaged mitochondria and is directly relevant to PD biology [115]. In SH-SY5Y cells and mouse brain, sustained OGA inhibition with Thiamet-G increased mitochondrial levels of PINK1 and microtubule-associated protein 1 light chain 3, whereas OGT knockdown or inducible OGT deletion reduced these markers; PINK1 was also shown to be O-GlcNAcylated [116]. However, these experiments were not performed in a PD model, did not identify the modified PINK1 residue, and did not establish Parkin recruitment or completion of mitophagic flux. These findings therefore support mechanistic plausibility rather than direct evidence that OGA inhibition restores PINK1–Parkin mitophagy in PD. Future studies should test this relationship in α-synuclein-driven and patient-derived dopaminergic-neuron models using flux-based assays and direct measurements of PINK1 stabilization, Parkin translocation, mitochondrial ubiquitination, and lysosomal delivery.

Neuroinflammation provides a second, potentially complementary therapeutic mechanism. A recent study integrated analyses of postmortem substantia nigra tissue from patients with PD, primary microglia, microglia-conditioned neuronal cultures, and an LPS-induced substantia nigra model [86]. Glucosamine and Thiamet-G increased microglial O-GlcNAcylation, reduced NF-κB- and NLRP3-related inflammatory signaling, and promoted anti-inflammatory and homeostatic marker profiles. In mice, Thiamet-G administered intraperitoneally at 20 mg/kg three times per week for four weeks improved motor performance, preserved tyrosine hydroxylase-positive neurons and striatal projections, and reduced glial activation. Complementary experiments using the 6-hydroxydopamine (6-OHDA) model showed that glucosamine supplementation or brain OGT overexpression attenuated motor deficits, dopaminergic neuronal loss, neuroinflammation, and mitochondrial dysfunction [85]. These findings support therapeutic interest while also defining the limits of the current evidence: LPS and 6-OHDA models reproduce inflammatory and dopaminergic injury components of PD but do not capture the full temporal progression or α-synuclein-driven biology of idiopathic disease.

PD-related studies suggest that O-GlcNAc signaling influences multiple pathogenic processes, including α-synuclein proteostasis, mitochondrial quality control, and microglial inflammatory responses. However, whether these mechanisms can be therapeutically coordinated through global, substrate-selective, or cell-type-specific intervention remains unresolved. Future studies should integrate disease-relevant models with temporal and cell-resolved analyses to define the most effective therapeutic strategy.

### 4.3. ALS/FTD Spectrum: Model-Based and Substrate-Focused Evidence

ALS and FTD comprise a clinically and biologically overlapping disease spectrum characterized by selective neuronal vulnerability, disruption of RNA and protein homeostasis, cytoskeletal abnormalities, oxidative stress, and, in many cases, pathological accumulation of TDP-43 [101,102,103,104]. Compared with AD and PD, evidence linking O-GlcNAcylation to the ALS/FTD spectrum remains less mature and is derived mainly from disease models and studies of selected substrates. The current literature therefore supports mechanistic plausibility rather than a unified disease-wide model.

In mutant superoxide dismutase 1 (SOD1) transgenic models, reduced protein O-GlcNAcylation has been reported in the nervous system [105]. This observation suggests that altered O-GlcNAc homeostasis may accompany motor-neuron disease processes, but it does not establish whether the change is an initiating driver, a compensatory response, or a downstream consequence of neurodegeneration. Longitudinal and cell-type-resolved studies are needed to clarify the temporal relationship between O-GlcNAc dysregulation and motor-neuron loss.

Neurofilaments represent a plausible substrate-level connection. Neurofilament proteins undergo extensive phosphorylation and are O-GlcNAcylated within domains relevant to cytoskeletal organization [106,107]. Given the importance of neurofilament abnormalities in motor-neuron disease, altered PTM balance may contribute to axonal dysfunction. However, direct evidence linking specific neurofilament O-GlcNAc sites to ALS progression remains limited, and mechanistic inference should be distinguished from established disease causality.

TDP-43 provides a more disease-proximal substrate. O-GlcNAcylation of TDP-43 has been reported to suppress pathological aggregation and improve RNA-splicing activity [110], suggesting that modification state can influence both proteostasis and functional activity of a central ALS/FTD-associated protein. This finding offers a potentially important site- or substrate-specific mechanism, although its relevance across disease stages, genetic backgrounds, and human tissues remains to be established.

Oxidative-stress pathways provide an additional possible connection. Studies involving glutathione peroxidase 7 (GPx7; also known as NPGPx) and O-GlcNAc cycling suggest that redox-stress sensing can interact with OGA-dependent regulation and influence cellular adaptation to oxidative injury [108,109]. Nevertheless, the extent to which these mechanisms specifically drive ALS/FTD pathology, rather than representing broader cellular stress responses, remains uncertain.

Evidence linking O-GlcNAcylation to ALS/FTD remains primarily substrate- and model-based. Studies should now determine whether modification of TDP-43, neurofilaments, and other disease-associated proteins directly contributes to their dysfunction or instead reflects secondary oxidative and proteostatic stress. Longitudinal, cell-resolved analyses will be essential to establish whether these changes precede protein mislocalization, aggregation, and neuronal injury.

### 4.4. Huntington’s Disease: Limited and Hypothesis-Generating Evidence for O-GlcNAc-Related Regulation

HD is caused by cytosine–adenine–guanine (CAG) trinucleotide repeat expansion in the huntingtin (HTT) gene and is characterized by progressive neuronal dysfunction, mutant huntingtin (mHTT) misfolding and aggregation, transcriptional abnormalities, impaired proteostasis, and disruption of nucleocytoplasmic transport [111,112]. Although O-GlcNAcylation has been proposed to intersect with several of these processes, the evidence base in HD remains limited and substantially less developed than that in AD or PD. Current findings are heterogeneous and do not yet establish a consistent direction of O-GlcNAc dysregulation or a unified therapeutic strategy.

One potential connection involves proteostasis and autophagic regulation. O-GlcNAc cycling can influence autophagy-related pathways in multiple cellular contexts [97,98], raising the possibility that altered O-GlcNAc states may affect mutant huntingtin turnover or aggregate handling. However, general autophagy findings do not establish an mHTT-specific mechanism, and the direction of the effect appears to vary with model, cell type, metabolic state, and intervention duration.

A second potential connection concerns nucleocytoplasmic transport. mHTT disrupts nuclear pore complex integrity and nucleocytoplasmic trafficking [112], and O-GlcNAcylation broadly regulates nuclear and cytoplasmic proteins. Nevertheless, direct evidence that disease-associated O-GlcNAc changes causally mediate nuclear pore dysfunction in HD is lacking. Evidence from hyperglycemia, diabetes-related signaling, or other metabolic-stress paradigms should likewise be treated as contextual rather than HD-specific.

Overall, the relationship between O-GlcNAcylation and HD remains hypothesis-generating. No disease-relevant O-GlcNAc-modified substrate has been reproducibly identified in human HD tissue or disease-relevant models. Priorities are therefore to establish reproducible O-GlcNAc alterations in human HD, determine their temporal relationship to mHTT accumulation and nucleocytoplasmic transport defects, and directly test candidate substrates rather than infer HD mechanisms from general effects on autophagy or metabolic stress.

Disease-specific O-GlcNAc-related evidence, mechanistic interpretations, and key references are summarized in Table 2.

## 5. Therapeutic Strategies Targeting O-GlcNAc Cycling

### 5.1. OGA Inhibitors: The Most Advanced Translational Strategy, but Not Yet a Validated Disease-Modifying Approach

Among available approaches, pharmacological OGA inhibition has progressed furthest toward clinical translation. Because OGA inhibitors elevate O-GlcNAcylation across a broad substrate landscape, they should be evaluated as dose- and exposure-dependent systems interventions rather than solely according to their effects on bulk O-GlcNAc abundance [3].

Acute Thiamet-G treatment altered more than 1200 transcripts in the normal mouse brain within 3 h, illustrating the pathway-wide biological effects of global OGA inhibition rather than disease-specific therapeutic efficacy [117]. Thiamet-G, the most widely used selective OGA inhibitor in mechanistic and preclinical studies, has reduced selected phospho-tau species and pathological tau accumulation in tauopathy models [43,44], modulated autophagy in a context-dependent manner [97,98], and attenuated inflammatory signaling and dopaminergic-neuron injury in an LPS-based PD model [86]. However, it remains primarily a research tool because its broad effects do not identify the specific substrates or cellular populations responsible for benefit or toxicity.

MK-8719 provides a more advanced preclinical example of brain-penetrant OGA inhibition [91]. Oral administration produced dose-dependent increases in O-GlcNAcylation in the brain and peripheral blood mononuclear cells, while positron emission tomography demonstrated central OGA target engagement in rats and rTg4510 mice. In the rTg4510 tauopathy model, MK-8719 reduced pathological tau accumulation and attenuated brain atrophy, including forebrain volume loss assessed by volumetric magnetic resonance imaging. These findings established the feasibility of integrating central exposure, peripheral pharmacodynamic measurements, imaging-based target engagement, and disease-relevant outcomes, while emphasizing the need to relate OGA occupancy to specific tau proteoforms and functional neurodegenerative processes.

Ceperognastat subsequently demonstrated that sustained, high central OGA occupancy could be achieved in humans. Phase 1 studies showed central nervous system penetration, acceptable short-term tolerability, and greater than 95% OGA enzyme occupancy, supporting dose selection for clinical efficacy testing [92]. However, the phase 2 PROSPECT-ALZ trial did not demonstrate clinical benefit in participants with early symptomatic AD [114]. The low-dose group showed no clinically meaningful slowing of disease progression, whereas the high-dose group exhibited greater clinical decline than the placebo group and a higher frequency of serious and severe treatment-emergent adverse events. Treatment was nevertheless associated with less whole-brain volume loss and modest changes in selected tau-related and plasma biomarkers. This biomarker–clinical dissociation demonstrates that central target engagement and apparently favorable changes in selected biomarkers are insufficient to predict therapeutic benefit.

Several explanations for the negative clinical outcome have been proposed, including excessive pathway engagement, a non-linear exposure–response relationship, disease-stage dependence, and unfavorable effects across the broader O-GlcNAc substrate network. These possibilities remain unproven and should be treated as testable hypotheses rather than established mechanisms. Future studies should therefore determine whether partial and near-complete OGA inhibition produce distinct effects on disease-relevant proteoforms, neuronal and glial states, synaptic function, clinical outcomes, and safety.

Future development should prioritize comparisons of chemically distinct OGA inhibitors to distinguish compound-specific liabilities from class-related effects. Studies should evaluate a broad range of central OGA occupancy, treatment initiation at different disease stages, chronic safety, reversibility, and regional and cell-type-resolved pharmacodynamics. Biomarker strategies should focus on correction of disease-relevant substrates, proteoforms, and cellular states rather than total O-GlcNAc elevation alone. Current evidence therefore establishes that OGA is pharmacologically accessible in the human central nervous system, but does not yet validate broad, sustained OGA inhibition as a disease-modifying treatment.

### 5.2. OGT and HBP Modulation: Upstream Control with Limited Selectivity

Targeting OGT or the hexosamine biosynthesis pathway (HBP) provides alternative upstream approaches for modulating O-GlcNAc signaling [8,9,10,11,12,13]. OGT catalyzes the transfer of GlcNAc from UDP-GlcNAc to protein substrates, whereas the HBP generates UDP-GlcNAc, the donor substrate for this reaction. Although these intervention points are mechanistically connected, they are not equivalent. OGT modulation directly alters the cellular capacity to install O-GlcNAc on protein substrates, whereas HBP manipulation affects a broader metabolic and biosynthetic network.

This distinction has important therapeutic implications. UDP-GlcNAc is not dedicated exclusively to nucleocytoplasmic O-GlcNAcylation but also supports N-linked and O-linked glycosylation, glycosaminoglycan synthesis, and other biosynthetic processes. Consequently, increasing HBP flux or precursor availability cannot be assumed to selectively enhance O-GlcNAcylation of protective substrates. Conversely, reducing HBP activity may disrupt broader glycosylation pathways and cellular metabolism independently of disease-relevant O-GlcNAc modifications. Moreover, changes in UDP-GlcNAc abundance do not necessarily produce proportional changes in protein O-GlcNAcylation because the final modification pattern is also shaped by OGT and OGA activity, substrate accessibility, subcellular compartmentalization, and compensatory feedback. HBP manipulation should therefore be regarded as a metabolic-network intervention rather than a selective O-GlcNAc-directed therapy.

Direct OGT modulation acts at the protein-modification step but presents a different selectivity challenge. OGT modifies a broad substrate repertoire and is required for essential cellular, developmental, and neuronal functions [24,25,26,71,72]. Global enhancement or inhibition of OGT activity may therefore simultaneously affect numerous signaling, transcriptional, metabolic, and stress-response pathways. The resulting effects are likely to depend on cell type, substrate availability, subcellular localization, magnitude of pathway modulation, and treatment duration. Accordingly, changes in total OGT activity cannot be assumed to produce uniform or therapeutically favorable changes across all substrates.

From an experimental perspective, perturbation of OGT and the HBP remains valuable for determining how metabolic inputs shape O-GlcNAc output and for identifying disease contexts in which upstream pathway dysregulation has functional consequences. From a therapeutic perspective, however, broad systemic manipulation faces substantial challenges, including limited central nervous system selectivity, peripheral metabolic and glycosylation effects, incomplete control over substrate-level responses, and potentially narrow safety windows.

At present, broad OGT and HBP modulation remains more useful for mechanistic investigation and target discovery than as an established precision therapeutic strategy. Substrate- and site-resolved approaches are discussed in Section 5.3.

### 5.3. Substrate- and Site-Resolved Approaches: Toward Precision O-GlcNAc Regulation

The major conceptual limitation of global O-GlcNAc modulation is that beneficial and detrimental effects may occur simultaneously across different substrates, cell types, subcellular compartments, and disease stages. A state-resolved strategy would instead seek to correct a disease-relevant O-GlcNAc state on a defined protein or modification site while preserving the broader O-GlcNAc network. Tau, α-synuclein, TDP-43, CREB, and Synapsin I illustrate why the relevant therapeutic or mechanistic unit may be a defined proteoform rather than total cellular O-GlcNAc abundance [43,44,45,46,52,53,94,96,99,110].

The dual-specificity RNA aptamer approach developed by Zhu and Hart provides an elegant proof of concept for substrate-selective O-GlcNAcylation [118]. The engineered RNA contains one recognition domain that binds OGT and a second that recognizes β-catenin, thereby recruiting endogenous OGT into proximity with the selected substrate. In the reported system, dual-specificity aptamers increased β-catenin O-GlcNAcylation without detectably increasing the modification of other OGT substrates examined. Coupling the platform to riboswitch elements or an inducible expression system further enabled temporal control. This work addresses a central challenge in the field—altering O-GlcNAcylation of a selected protein without broadly perturbing the O-GlcNAc proteome—and provides a valuable platform for establishing substrate-level causality.

The demonstrated selectivity of this approach is substrate-directed rather than cell- or site-specific. Cell specificity would require restricted expression or delivery to a defined neuronal or glial population, whereas site specificity would require additional control over which serine or threonine residue is modified. Translation to neurodegenerative disease would also require adequate RNA stability, efficient intracellular delivery, blood–brain barrier penetration, cell-type-restricted uptake or expression, control of the magnitude and duration of activity, avoidance of innate immune activation, minimal off-target binding, scalable manufacturing, and acceptable long-term safety. Because the two aptamer domains must retain their independent binding functions within a single RNA construct, intracellular folding, linker architecture, relative binding affinities, and expression level may also influence substrate recruitment and modification efficiency.

The near-term value of this platform is nevertheless substantial. Aptamer-directed O-GlcNAcylation can determine whether increasing O-GlcNAcylation of a selected disease-associated protein is sufficient to alter a cellular phenotype. When combined with complementary loss-of-function approaches—such as substrate-directed O-GlcNAc removal, disruption of OGT recruitment, or carefully controlled endogenous site perturbation—it may also help establish whether the relevant modification is necessary. Because mutation of a serine or threonine residue can simultaneously alter phosphorylation, protein conformation, and neighboring PTM networks, site-mutant phenotypes should not be attributed specifically to loss of O-GlcNAcylation without orthogonal biochemical confirmation and appropriate phospho-state controls. Application to tau, α-synuclein, or TDP-43 in neurons, microglia, and multicellular disease models could distinguish substrate-specific effects from the broader consequences of global OGA inhibition and identify targets that warrant further therapeutic development. A staged experimental pathway should begin with confirmation of endogenous target engagement, quantitative mapping of the modified site or sites, and demonstration of functional reversibility using orthogonal gain- and loss-of-function approaches in cells; proceed to inducible and cell-restricted expression in brain-relevant models; and subsequently evaluate delivery systems capable of supporting controlled and sustained activity in vivo.

Other proximity-based bifunctional systems, ligand-directed chimeras, genetic recoding, and synthetic or semisynthetic glycoproteoform approaches provide complementary routes toward substrate or site resolution [119,120,121,122]. These technologies differ substantially in their mechanisms, intended applications, and developmental maturity. Some primarily serve as chemical-biology tools for establishing causality, whereas others may constitute early therapeutic prototypes. Candidate substrates and sites should be prioritized only when disease-associated alteration, causal functional relevance, the required direction and magnitude of correction, and an appropriate cell-type and disease-stage window have been established.

Substrate- and site-resolved technologies should therefore be developed in parallel with the continued evaluation of brain-penetrant OGA inhibitors rather than being treated as a prerequisite for all clinical investigation. Their immediate roles are to identify causal proteoforms, determine which substrates mediate the benefits or liabilities of broad pathway modulation, interpret positive or negative pharmacological results, and nominate targets for next-generation interventions. Their eventual therapeutic value will depend on integrating molecular selectivity with brain delivery, cell-type restriction, reversible or temporally controlled activity, and pharmacodynamic assays capable of measuring correction of the intended proteoform.

### 5.4. Combination Strategies: Mechanistic Rationale and Translational Uncertainty

Neurodegenerative diseases arise from interacting pathological processes rather than isolated molecular defects. O-GlcNAc signaling intersects with protein aggregation, autophagy–lysosome function, mitochondrial homeostasis, synaptic regulation, inflammatory responses, and metabolic adaptation [19,20,70]. This broad network involvement provides a rationale for combining O-GlcNAc-directed interventions with therapies that target complementary disease mechanisms.

Potential combinations could pair O-GlcNAc modulation with interventions directed against pathological protein aggregation, impaired autophagy–lysosome function, mitochondrial dysfunction, or neuroinflammation. In principle, combination strategies could permit lower exposure or more limited O-GlcNAc pathway modulation while retaining disease-relevant benefit. For example, a substrate-selective O-GlcNAc intervention could be combined with a treatment targeting a distinct downstream pathological process, whereas partial OGA inhibition could be paired with a non-overlapping disease-modifying mechanism.

Because O-GlcNAc signaling influences autophagy, metabolism, inflammation, synaptic function, and proteostasis, combination partners may produce additive, synergistic, antagonistic, or unexpected network effects. Future studies should therefore include appropriate monotherapy and combination groups, formal analysis of pharmacological interaction, and measurements that distinguish target engagement from functional benefit. Dose, treatment sequence, duration, disease stage, cell-type-specific effects, and pharmacokinetic or pharmacodynamic interactions should be evaluated to determine whether a combination improves efficacy, permits dose reduction, widens the therapeutic window, or limits toxicity relative to either treatment alone.

Combination therapy should therefore be regarded as a promising but exploratory translational direction rather than an established O-GlcNAc therapeutic strategy. Its value will depend on demonstrating that mechanistically complementary interventions provide benefits beyond those achieved by either treatment alone without introducing unacceptable network-level toxicity.

The core value, key limitations, and current developmental status of each therapeutic strategy are outlined in Table 3.

## 6. Discussion

The available evidence does not support a universal model in which increasing or decreasing total O-GlcNAcylation is uniformly beneficial across neurodegenerative diseases. Instead, the functional consequences of O-GlcNAc modulation depend on the biological state being altered, including the disease context, affected cell population, subcellular compartment, disease-relevant substrate and modification site, and timing and magnitude of intervention. This state-resolved framework helps reconcile findings that might otherwise appear contradictory. In tauopathy models, pharmacological OGA inhibition can reduce selected phospho-tau species and improve disease-related phenotypes, whereas site-specific O-GlcNAcylation of α-synuclein can alter aggregation, conformational behavior, and seeding without necessarily corresponding to a uniform change in total cellular O-GlcNAc abundance. Similarly, interactions between O-GlcNAc cycling, autophagy, mitochondrial adaptation, and inflammatory signaling vary according to basal metabolic state, cell type, and experimental duration. Thus, bulk O-GlcNAc abundance should be regarded as an initial indicator rather than a sufficient mechanistic endpoint. Mechanistic and therapeutic interpretation requires identification of the relevant cell type, substrate, modification site, and intervention window.

The maturity of the evidence base differs substantially among neurodegenerative diseases. AD and related tauopathies currently show the strongest convergence of human tissue observations, mechanistic models, pharmacological intervention, and clinical-stage pathway targeting. PD is supported by substantial substrate-level and disease-model evidence, particularly regarding α-synuclein proteoform behavior, dopaminergic vulnerability, and microglial inflammation. Evidence linking O-GlcNAcylation to PINK1-dependent mitophagy is mechanistically suggestive but has not yet been validated in PD-specific models, and direct clinical evidence remains absent. Evidence in the ALS/FTD spectrum is more fragmented and is derived mainly from experimental models involving cytoskeletal regulation, TDP-43 proteostasis, RNA processing, and cellular stress responses. In HD, the proposed links between O-GlcNAcylation, mutant huntingtin handling, autophagy, and nucleocytoplasmic transport remain largely hypothesis-generating. These differences argue against extrapolating therapeutic conclusions from one disease to another and highlight the need to align future studies with the maturity and specific limitations of each evidence base.

The distinction between association and causality is especially important. A disease-associated reduction or elevation in O-GlcNAcylation may represent an initiating mechanism, an adaptive or compensatory response, or a downstream consequence of altered metabolism, inflammation, or cellular composition. Cross-sectional measurements of total O-GlcNAc abundance cannot distinguish among these possibilities. Even detection of a site-specific alteration does not establish functional relevance unless selective perturbation of that modification produces a reproducible phenotypic effect.

Stronger causal inference will require longitudinal sampling, endogenous site perturbation, cell-type-specific genetic or chemical manipulation, disease-stage-resolved experiments, and rescue designs that distinguish direct substrate effects from secondary consequences of global pathway modulation. Three operational criteria may be useful for prioritizing candidate O-GlcNAc events. First, the modification should show a reproducible temporal or disease-associated pattern, ideally preceding or predicting relevant pathology in longitudinal models or human samples. Second, selective perturbation of the substrate or site should produce a reproducible functional effect while leaving total cellular O-GlcNAcylation largely unchanged. Third, the phenotype should be reversible through restoration of the relevant modification state and supported by orthogonal evidence, such as human pathology, longitudinal biomarker data, genetic findings, or convergent results across independent models. These criteria should be regarded as complementary rather than mandatory in every case, because the absence of human genetic evidence does not by itself exclude a causal role for a specific O-GlcNAc event.

Global pathway modulation alters a broad substrate network involving synaptic proteins, transcription factors, metabolic enzymes, inflammatory regulators, mitochondrial proteins, and proteostasis machinery. Its net effect therefore reflects multiple potentially concordant or opposing responses rather than correction of a single pathological mechanism. A precision framework should instead define which disease-relevant O-GlcNAc state requires correction, in which cell type and compartment, at which substrate or site, and during which disease stage.

The negative PROSPECT-ALZ trial illustrates this distinction. High central OGA occupancy and selected biomarker changes did not produce clinical benefit, while the higher dose was associated with greater clinical decline than placebo [114]. These findings do not exclude disease-relevant roles for specific O-GlcNAc events; rather, they indicate that broad pathway engagement may modify substrates associated with both benefit and liability. Candidate interventions should therefore be prioritized using convergent evidence of reproducible disease-associated alteration, functional effects after selective perturbation, mechanistic separation from bulk O-GlcNAc changes, reversibility, and compatibility with a realistic therapeutic window.

Methodological limitations remain a major barrier to state-resolved analysis. O-GlcNAc modifications are dynamic, often substoichiometric, and difficult to preserve, enrich, localize, and quantify reproducibly [61,62,63]. Bulk brain-tissue measurements may also obscure opposing changes across cell populations. Spatially and cell-type-resolved profiling, integrated where appropriate with phosphoproteomics, transcriptomics, metabolomics, imaging, and functional phenotyping, will therefore be required to distinguish disease-relevant modifications from secondary metabolic or stress-associated changes.

Human validation and pharmacodynamic measurement are equally important. Mechanistic findings from cell lines, toxin models, transgenic animals, and chemically defined protein systems should be evaluated using postmortem tissue, patient-derived cells, organoids, multicellular models, cerebrospinal fluid, extracellular vesicles, and longitudinal samples where feasible. Substrate- or site-directed technologies will have translational value only if they achieve adequate brain and intracellular delivery, molecular stability, cell-type restriction, reversible control, and long-term safety. Corresponding biomarkers must demonstrate correction of the intended disease-relevant modification state at an exposure associated with functional benefit, rather than target engagement or total O-GlcNAc elevation alone.

## 7. Conclusions

O-GlcNAcylation regulates neuronal and glial processes implicated in neurodegeneration, but its consequences depend on disease, cell type, substrate, subcellular compartment, metabolic state, and intervention timing. Current evidence therefore does not support total O-GlcNAc abundance as a uniform indicator of protection or toxicity. Translational evidence is most advanced in AD and related tauopathies, remains predominantly preclinical in PD, and is more limited or hypothesis-generating in ALS/FTD and HD.

Therapeutic development should move from indiscriminate pathway elevation or suppression toward correction of defined disease-relevant O-GlcNAc states. The negative ceperognastat trial demonstrates that central target engagement and selected biomarker changes are insufficient without functional benefit and acceptable safety. Future studies should prioritize causal substrate- and site-resolved analysis, cell-type-specific pharmacodynamics, exposure and disease-stage dependence, and biomarkers that link correction of the intended modification state to functional outcomes. Whether global OGA inhibition remains viable within a narrower therapeutic window requires direct experimental and clinical demonstration.

## Figures and Tables

**Table 1:** Representative brain cell types and subcellular compartments, O-GlcNAc-related substrates and pathways, functional implications, and evidence boundaries.

Cell Type or Compartment	Representative Substrates or Pathways	Main Functional Implications	Evidence Characteristics and Boundaries	Key References
Presynaptic and axonal compartments	Synapsin I, eukaryotic translation initiation factor 4 gamma (eIF4G), Milton	Synaptic-vesicle organization, local axonal translation, mitochondrial trafficking, and activity–energy coupling	Synapsin I includes site-resolved functional evidence; eIF4G and Milton provide mechanistic evidence for axonal translation and mitochondrial transport. Generalization to all presynaptic proteins is not warranted.	[27,28,45,46,57,58,59]
Postsynaptic receptor-associated regulation	GluA2, GluN1, OGT-dependent synaptic pathways	Receptor-associated plasticity, long-term depression (LTD)-related regulation, excitatory-synapse maturation, and disease-associated synaptic dysfunction	Functional evidence is available for selected receptor-associated mechanisms, but the strength and disease relevance differ among substrates and models.	[47,48,49,54,64]
postsynaptic density (PSD) organization and phase separation	synaptic GTPase-activating protein (SynGAP)/postsynaptic density protein 95 (PSD-95) system; SH3 and multiple ankyrin repeat domains protein (SHANK), guanylate kinase-associated protein (GKAP), and Homer as structural context	Organization of postsynaptic signaling assemblies and phase-separation-related regulation	Direct evidence supports O-GlcNAc-dependent regulation of SynGAP/PSD-95 phase behavior. SHANK, GKAP, and Homer primarily provide structural context unless direct O-GlcNAc-dependent functions are independently demonstrated.	[60,61,62,63,77,78,79,80,81,82,83]
Nuclear transcriptional regulation	cAMP response element-binding protein (CREB); brain and muscle ARNT-like protein 1 (BMAL1)/circadian locomotor output cycles kaput (CLOCK)-related pathways; developmental signaling networks	Memory-related transcription, circadian regulation, neurodevelopment, and stress responses	Selected substrates have direct mechanistic evidence, whereas broader pathway-level effects remain dependent on cell type and developmental or disease context.	[50,51,52,53,55,56,70,73]
Mitochondrial and mitochondria-associated regulation	ATP synthase F1 subunit alpha (ATP5A), Milton, and activity-responsive O-GlcNAc pathways	Bioenergetic regulation, mitochondrial trafficking, neuronal energy adaptation, and oxidative-stress responses	Mitochondrial protein modification should be distinguished from mitochondria-associated regulation and indirect cytosolic effects on mitochondrial function.	[27,28,65]
Glial cells	nuclear factor kappa B (NF-κB)-related and inflammasome-associated pathways; microglial O-GlcNAc programs	Inflammatory-state regulation, immune responses, and modulation of the neuronal microenvironment	Emerging cell-type-specific evidence is strongest in selected microglial models; bulk brain analyses may obscure opposing responses among glial and neuronal populations.	[84,85,86]

**Table 2:** Disease-specific O-GlcNAc-related evidence, representative models and agents, and mechanistic interpretations.

Disease	Representative Evidence, Models, and Agents	Mechanistic Interpretation	Key References
AD/tauopathy	Postmortem human hippocampus; JNPL3 and rTg4510 tauopathy models; intracerebroventricular lipopolysaccharide (LPS)-induced neuroinflammation model; primary microglia; Thiamet-G, MK-8719, ceperognastat, glucosamine administered at 200 mg/kg (i.p.), three times weekly for 4 weeks	Tau PTM regulation, aggregation, and clearance; APP processing; mitochondrial ATP synthase function; microglial NF-κB/NLRP3-associated inflammatory regulation	[39,40,41,42,43,44,65,84,87,88,89,90,91,92,114]
PD/α-synucleinopathy	Postmortem human substantia nigra; 6-hydroxydopamine (6-OHDA) and LPS-induced mouse models; primary microglia and microglia-conditioned neuronal cultures; human induced pluripotent stem cell (iPSC)-derived dopaminergic neurons; chemically defined α-synuclein O-GlcNAc proteoforms; Thiamet-G (20 mg/kg, i.p., 3×/week, 4 weeks), glucosamine, OGT overexpression	α-Synuclein aggregation, conformational strain, seeding, and propagation; dopaminergic-neuron survival; mitochondrial quality-control pathways, with a putative link to PTEN-induced kinase 1 (PINK1)-associated mitophagy that remains to be validated in PD-relevant models; context-dependent autophagic and lysosomal regulation; microglial inflammatory signaling	[85,86,93,94,95,96,97,98,99,100,115,116]
ALS/FTD spectrum	Mutant SOD1 transgenic models; neurofilament PTM and O-GlcNAc studies; TAR DNA-binding protein 43 (TDP-43) O-GlcNAcylation and RNA splicing assays; glutathione peroxidase 7 (GPx7) oxidative-stress models	Cytoskeletal organization and axonal integrity; TDP-43 proteostasis and RNA splicing; redox-stress adaptation	[101,102,103,104,105,106,107,108,109,110]
HD	Limited cellular and pathway-level evidence involving mutant huntingtin (mHTT) proteostasis, autophagy-related processes, stress responses, and nucleocytoplasmic transport.	Potential effects on aggregate handling, proteostasis, cellular stress responses, and nucleocytoplasmic transport; however, the evidence remains hypothesis-generating, and direct disease-relevant O-GlcNAc substrates and modification sites have not been established.	[111,112,113]

**Table 3:** Therapeutic strategies targeting O-GlcNAc signaling: representative approaches, potential value, major limitations, and developmental status.

Strategy	Representative Approach	Potential Value	Major Limitations	Evidence and Developmental Status	Key References
OGA inhibitors	Thiamet-G; MK-8719; ceperognastat; other brain-penetrant OGA inhibitors	Represents the most clinically advanced approach to increasing O-GlcNAcylation; enables measurable central target engagement; has shown preclinical effects on pathological tau, brain atrophy, proteostasis, and inflammatory responses	Broad effects across substrates and cell types; potentially non-linear or narrow therapeutic window; possible dissociation between biomarker changes and clinical outcomes; uncertain consequences of sustained synaptic, metabolic, and systemic pathway modulation	Supported by mechanistic studies, multiple preclinical models, phase 1 studies, and phase 2 clinical testing. Clinical benefit has not been demonstrated. In the PROSPECT-ALZ trial, high-dose ceperognastat was associated with greater clinical decline than placebo and a greater frequency of severe or serious adverse events than placebo	[43,44,86,91,92,114]
OGT modulation	Pharmacological or genetic alteration of OGT activity; inducible, spatially restricted, or cell-type-selective OGT manipulation	Directly regulates the addition of O-GlcNAc to protein substrates; useful for defining causal pathway effects and identifying disease-relevant substrates and cellular states	OGT is essential and modifies a broad substrate repertoire; limited substrate and cell-type selectivity; potential effects on development, transcription, metabolism, stress responses, and neuronal function	Primarily mechanistic and preclinical; no established clinical strategy	[24,25,26,71,72]
HBP modulation	Glucosamine or precursor supplementation; manipulation of HBP enzymes, nutrient inputs, substrate availability, or metabolic flux	Connects metabolic and nutrient signals to UDP-GlcNAc availability and O-GlcNAc output; may identify metabolically responsive disease states	Not selective for nucleocytoplasmic O-GlcNAcylation; affects broader glycosylation and biosynthetic pathways; UDP-GlcNAc abundance does not translate linearly into substrate-specific O-GlcNAcylation; potential peripheral metabolic effects	Mechanistic and preclinical; currently more useful for pathway interrogation than for precision therapy	[8,9,10,11,12,13,84,85,86]
Substrate-selective O-GlcNAc targeting	Dual-specificity RNA aptamers; proximity-based bifunctional systems; nanobody–enzyme fusions; ligand-directed chimeras	Enables preferential modification of a selected substrate; supports substrate-level causal analysis; may distinguish beneficial from detrimental substrates affected by global pathway modulation	Not intrinsically site- or cell-specific; challenges include intracellular delivery, blood–brain barrier access, RNA or protein stability, molecular folding, off-target binding, immune activation, activity control, manufacturing, and long-term safety	Early proof-of-concept and chemical-biology development. Dual-specificity RNA aptamers have selectively increased β-catenin O-GlcNAcylation in cellular systems	[118,119,122]
Site-resolved tools and defined glycoproteoforms	Genetic recoding; synthetic or semisynthetic O-GlcNAc proteoforms; modification-site mutants; site-resolved chemical biology	Defines the causal effects of individual modification sites; identifies disease-relevant proteoforms; supports target validation and development of proteoform-specific pharmacodynamic biomarkers	Technically demanding and predominantly research-oriented; limited compatibility with chronic in vivo delivery; experimentally generated proteoforms may not fully reproduce endogenous occupancy, localization, or cellular context	Established or emerging mechanistic tools; currently provide proof of site-specific function rather than direct therapeutic platforms	[94,96,99,120,121]
Combination strategies	Partial OGA inhibition combined with anti-aggregation, autophagy–lysosome, mitochondrial, or anti-inflammatory interventions; substrate-selective modulation combined with a complementary downstream therapy	May enable lower exposure or partial pathway modulation; could widen the therapeutic window and address interacting pathological processes through complementary mechanisms	Therapeutic synergy has not been demonstrated; possible additive toxicity, antagonism, pathway overlap, compensatory effects, and pharmacokinetic or pharmacodynamic drug–drug interactions	Hypothesis-generating and predominantly preclinical; requires comparison with each monotherapy and formal evaluation of pharmacological interactions	[3,11,12,19,20,70]

## Data Availability

No new data were created or analyzed in this study. Data sharing is not applicable to this article.

## Abbreviations

The following abbreviations are used in this manuscript:

AD	Alzheimer’s disease
ALS	Amyotrophic lateral sclerosis
AMPA	α-Amino-3-hydroxy-5-methyl-4-isoxazolepropionic acid
APP	Amyloid precursor protein
ATP	Adenosine triphosphate
ATP5A	ATP synthase F1 subunit alpha
BMAL1	Brain and muscle ARNT-like protein 1
CaMKII	Calcium/calmodulin-dependent protein kinase II
CAG	Cytosine–adenine–guanine trinucleotide repeat
CLOCK	Circadian locomotor output cycles kaput
CREB	cAMP response element-binding protein
eIF4G	eukaryotic translation initiation factor 4 gamma
FTD	Frontotemporal dementia
GKAP	Guanylate kinase-associated protein
GPx7/NPGPx	glutathione peroxidase 7
HBP	Hexosamine biosynthesis pathway
HD	Huntington’s disease
hnRNP R	heterogeneous nuclear ribonucleoprotein R
HTT	huntingtin
iPSC	Induced pluripotent stem cell
mHTT	Mutant huntingtin
LTD	Long-term depression
LPS	Lipopolysaccharide
mTOR	Mechanistic target of rapamycin
NF-κB	Nuclear factor kappa B
NLRP3	NOD-like receptor family pyrin domain-containing 3
NMDA	N-methyl-D-aspartate
NMDAR	N-methyl-D-aspartate receptor
NPC	nuclear pore complex
O-GlcNAc	O-linked β-N-acetylglucosamine
OGA	O-GlcNAcase
OGT	O-GlcNAc transferase
PD	Parkinson’s disease
PSD	Postsynaptic density
PSD-95	Postsynaptic density protein 95
p-tau	phosphorylated tau
PTM	Post-translational modification
PINK1	PTEN-induced kinase 1
RNA	Ribonucleic acid
SHANK	SH3 and multiple ankyrin repeat domains protein
SOD1	Superoxide dismutase 1
STAT3	Signal transducer and activator of transcription 3
SynGAP	synaptic GTPase-activating protein
TDP-43	TAR DNA-binding protein 43
UDP-GlcNAc	Uridine diphosphate β-N-acetylglucosamine
UTP	Uridine triphosphate
6-OHDA	6-Hydroxydopamine
α-Syn	α-synuclein
